# Domain-general subregions of the medial prefrontal cortex contribute to recovery of language after stroke

**DOI:** 10.1093/brain/awx134

**Published:** 2017-06-27

**Authors:** Fatemeh Geranmayeh, Tsz Wing Chau, Richard J. S. Wise, Robert Leech, Adam Hampshire

**Affiliations:** Computational Cognitive and Clinical Neuroimaging Laboratory, Imperial College, Hammersmith Hospital Campus, Du Cane Road, London, W12 0NN, UK

**Keywords:** aphasia, stroke, longitudinal recovery, cingular-opercular network, domain-general

## Abstract

We hypothesized that the recovery of speech production after left hemisphere stroke not only depends on the integrity of language-specialized brain systems, but also on ‘domain-general’ brain systems that have much broader functional roles. The presupplementary motor area/dorsal anterior cingulate forms part of the cingular-opercular network, which has a broad role in cognition and learning. Consequently, we have previously suggested that variability in the recovery of speech production after aphasic stroke may relate in part to differences in patients’ abilities to engage this domain-general brain region. To test our hypothesis, 27 patients (aged 59 ± 11 years) with a left hemisphere stroke performed behavioural assessments and event-related functional magnetic resonance imaging tasks at two time points; first in the early phase (∼2 weeks) and then ∼4 months after the ictus. The functional magnetic resonance imaging tasks were designed to differentiate between activation related to language production (sentential overt speech production—Speech task) and activation related to cognitive processing (non-verbal decision making). Simple rest and counting conditions were also included in the design. Task-evoked regional brain activations during the early and late phases were compared with a longitudinal measure of recovery of language production. In accordance with a role in cognitive processing, substantial activity was observed within the presupplementary motor area/dorsal anterior cingulate during the decision-making task. Critically, the level of activation within this region during speech production correlated positively with the longitudinal recovery of speech production across the two time points (as measured by the in-scanner performance in the Speech task). This relationship was observed for activation in both the early phase (*r* = 0.363, *P* = 0.03 one-tailed) and the late phase (*r* = 0.538, *P* = 0.004). Furthermore, presupplementary motor area/dorsal anterior cingulate activity was a predictor of both language recovery over time and language outcome at ∼4 months, over and above that predicted by lesion volume, age and the initial language impairment (general linear model overall significant at *P < *0.0001; ExpB 1.01, *P* = 0.02). The particularly prominent relationship of the presupplementary motor area/dorsal anterior cingulate region with recovery of language was confirmed in voxel-wise correlation analysis, conducted unconstrained for the whole brain volume. These results accord with the hypothesis that the functionality of the presupplementary motor area/dorsal anterior cingulate contributes to language recovery after stroke. Given that this brain region is often spared in aphasic stroke, we propose that it is a sensible target for future research into rehabilitative treatments. More broadly, baseline assessment of domain-general systems could help provide a better prediction of language recovery.

## Teaser

The pre-supplementary motor area/dorsal anterior cingulate (SMA/dACC) has a broad role in cognition. Geranmayeh *et al.* perform longitudinal fMRI in 27 patients after stroke, and show that preSMA/dACC activity during speech production positively correlates with levels of spontaneous recovery. Recruitment of domain-general systems can thus help to restore language function.

## Introduction

Language processing is arguably our most sophisticated ability and its impairment after aphasic stroke is both common and debilitating. Consequently, the mechanisms of language recovery after aphasic stroke have been the focus of much debate in the literature (Price and Crinion, 2005; Crinion and Leff, 2007; Geranmayeh *et al.*, 2014*a*).

A large proportion of previous studies on this topic have focused on relating recovery to the structure and function of perilesional regions that are classically considered to have language-specialized functions (Heiss *et al.*, 1999; Warburton *et al.*, 1999; Hillis *et al.*, 2006; Meinzer and Breitenstein, 2008; Szaflarski *et al.*, 2013). The remainder have focused on understanding the involvement of contralateral homotopic regions in the right hemisphere (Musso *et al.*, 1999; Blasi *et al.*, 2002; Leff *et al.*, 2002; Winhuisen *et al.*, 2005; Saur *et al.*, 2006; Turkeltaub *et al.*, 2012), which is believed to facilitate recovery, or the contralesional transcallosal disinhibition of the lesioned hemisphere, which is believed to hinder recovery (Belin *et al.*, 1996; Blank *et al.*, 2003; Naeser *et al.*, 2004, 2005; Thiel *et al.*, 2006).

More recently, it has been proposed that ‘domain-general’ brain networks also play a key role in recovery from aphasic stroke. This hypothesis is based on the assertion that language performance is not only dependent on brain regions that display language-specialized functions such as phonological, semantic, and syntactic processing (Indefrey and Levelt, 2004; Hickok and Poeppel, 2007; Rauschecker and Scott, 2009; Fedorenko *et al.*, 2011; Price, 2012), but also on widely distributed and often overlapping brain regions that make broader, or ‘domain-general’, contributions to cognition (Fedorenko, 2014; Fedorenko and Thompson-Schill, 2014; Geranmayeh *et al.*, 2014a,*b*). Indeed, the area of cortex within the classical Broca’s area contains subregions that have both of these properties (Fedorenko *et al.*, 2012, 2013).

These domain-general regions support a variety of processes including working memory, reasoning, attention, and executive function (Duncan and Owen, 2000; Fedorenko *et al.*, 2013). One specific domain-general network that has been suggested to support recovery of language function after stroke is the cingular-opercular network. This network includes the dorsal anterior cingulate cortex, the adjacent presupplementary motor area (preSMA/dACC) and the bilateral anterior insular with adjacent inferior frontal gyrus These brain regions lie within the so-called Multiple Demand Cortex (Fig. 3B, in yellow), a widely distributed volume of cortex that is commonly co-recruited and supports a variety of cognitive processes (Duncan and Owen, 2000; Duncan, 2010; Fedorenko *et al.*, 2013).

Activation of the brain regions within the cingular-opercular network has been associated with a particularly wide variety of tasks that require the wilful control of behaviour, including inhibitory control (Hampshire and Sharp, 2015), target detection (Hampshire *et al.*, 2007), detection of salient events (Seeley *et al.*, 2007; Menon and Uddin, 2010; Power *et al.*, 2011; Power and Petersen, 2013), attentional switching (Hampshire and Owen, 2006), and the attentional control required for stable task set maintenance (Dosenbach *et al.*, 2007, 2008; Power *et al.*, 2011; Power and Petersen, 2013). Most relevant to recovery of behaviour after stroke, is the increased activity of the cingular-opercular network when new behaviours are being learnt. These include the learning of new visuomotor associations (Doyon *et al.*, 1996; Toni *et al.*, 2001), stimulus response rules (Hampshire *et al.*, 2016) and, critically learning a pseudo-language (Sliwinska *et al.*, submitted for publication). Based on the broad role of the cingular-opercular network in learning and cognition, we have previously proposed that the integrity of its function, in particular in the preSMA/dACC, may underlie a better language outcome after aphasic stroke (Brownsett *et al.*, 2014; Geranmayeh *et al.*, 2014*a*).

Here we tested a direct prediction of this hypothesis in a functional MRI study that was designed to investigate the relationship between cingular-opercular network activity, and the longitudinal recovery of speech production after stroke. Specifically, a cohort of patients undertook event-related functional MRI tasks first at ∼2 weeks (early scan) and then at ∼4 months (late scan) after a left hemisphere stroke. This is a time at which the most rapid recovery typically occurs. The functional MRI tasks were carefully chosen to differentiate between regions of brain activity that related to language processes (namely sentential overt speech production) or to cognitive processes more generally (e.g. non-verbal decision making). In accordance with our hypotheses, we focused our analyses on the preSMA/dACC region; however, we also confirmed the prominent role of this region by applying voxel-wise analyses to the whole brain volume. Our prediction was that the magnitude of activation within this domain-general brain region would correlate positively with the longitudinal recovery of speech production after a left hemisphere stroke.

## Materials and methods

### Participants

**Figure 1:**
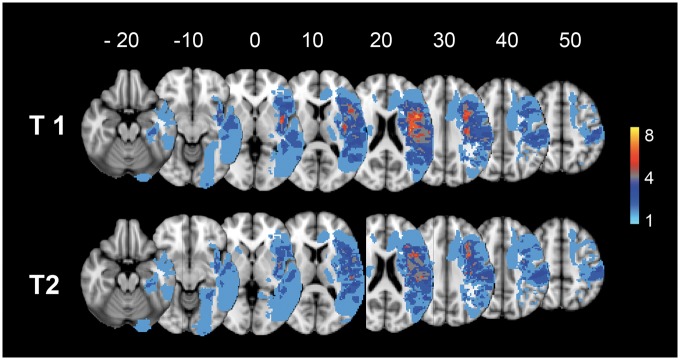
**Overlay of the lesion distribution in the patients with left hemisphere stroke, projected on standard brain templates.** Lesions were defined separately at each time point. The colour code represents the number of patients with a lesion in a given voxel. The numbers above the brain slices represent the MNI coordinates in the *z* plane, in millimetres. T_1_: ∼2 weeks after stroke; T_2_: ∼4 months after stroke. Lesion volumes were highly correlated across both sessions (*r* = 0.969, *P* < 0.001) but they were lower in the second session reflecting post-stroke atrophy of the surrounding tissues. The values derived from the first session were used in the analyses and are listed in Supplementary Table 1.

The National Research Ethics Service Committee approved this study. Patients with left hemisphere infarcts and premorbid fluency in English were recruited. Patients with a previous history of a stroke resulting in aphasia or other neurological illness, or concurrent use of psychoactive drugs, were excluded. Twenty-seven participants were scanned at two time points; first in the early phase after the stroke [15 ± 7.6 days, mean ± standard deviation (SD)] and again at ∼4 months (108 ± 26 days) after an average interscan interval of 93 ± 26 days. The patients were aged 59.1 ± 10.8 years with a male:female ratio of 1:0.6. One patient was left-handed, but was clearly aphasic at stroke onset. All patients had infarcts in the left hemisphere. The average lesion volume was 41.4 ± 44.4 cm^3^ (Fig. 1). Three patients had additional small infarcts (0.5, 1.5 and 3.5 cm^3^) in the right hemisphere. In two cases this right-sided lesion predated that of the infarct in the left hemisphere without causing any lasting clinical deficits. Demographic information, details of the stroke, and assessments are provided in Supplementary Table 1.

Twenty-four age-matched, right-handed, fluent English-speaking healthy participants were also recruited (aged 57 ± 11 years; female ratio = 1:4). Data from controls and cross-sectional analyses of patient data have been previously published (Geranmayeh *et al.*, 2014*b*, 2015*a*, 2016). Only the behavioural results from controls will be discussed in this article (Table 1).

**Table 1:** In-scanner performance and behavioural assessments

	**Healthy controls**	**Patients T_1_**	**Patients T_2_**
	**(*n* = 24)**	**(*n* = 27)**	**(*n* = 27)**
**In-scanner performance**			
Language measure	7.6 ± 1.6	3.6 ± 2.6**	4.7 ± 1.8^§§^
Syllable rate (syllable/s)	2.7 ± 0.6	1.5 ± 0.7**	1.7 ± 0.6^§^
Decision task (% correct responses)	1 ± 0.04	0.95 ± 0.1	0.98 ± 0.0
Decision task (mean reaction time, ms)	0.37 ± 0.1	0.43 ± 0.1*	0.40 ± 0.1^§§^
**Assessments**			
Cognitive score (38)	37.7 ± 0.6	33.8 ± 4.7**	36.7 ± 1.8^§^
Verbal fluency (semantic and phonetic)	42.9 ± 9.8	16.7 ± 13.6**	28.5 ± 12.5^§§^
Ravens Matrix (12)	11.7 ± 0.5	10.1 ± 2.2*	11.0 ± 1.7^§§^
Spontaneous Speech	25.9 ± 2.5	13.0 ± 7.5**	18.7 ± 6^§§^
Comprehension of Written language (53/62)		47.9 ± 15.6	58.1 ± 5.1^§§^
Comprehension of Spoken language (56/62)		55.0 ± 11.5	62.4 ± 4.2^§§^
Repetition (67/74)		56.9 ± 19.1	68.2 ± 6.8^§^
Object naming (51/58)		44.5 ± 15.4	54.7 ± 4.7^§§^
Reading (58/70)		50.3 ± 24.4	64.1 ± 8.1^§§^
Spoken picture description (>33)		52.3 ± 33.4	79.0 ± 36.8^§§^

Numbers in brackets accompanying the assessments refer to (aphasia ‘cut-off’ score / maximum possible score). Data are indicated as mean ± SD.

*P < 0.05 and **P < 0.001 refer to independent samples *t-*test between controls and patients at T_1_ (early scan).

^§^*P* < 0.05 and ^§§^*P* < 0.001 refer to paired sample *t-*test in patients at both time points. All the assessments listed in the bottom half of the table are subsets of the CAT battery, except for Ravens Matrix and Spontaneous Speech (see text for details).

### Behavioural assessments

The key measure of speech production ability was the in-scanner language measure henceforth called ‘language measure’ (Table 1). During the scan, speech production was recorded using a magnetic resonance compatible microphone (Optoacoustics FOMRI-III). These were transcribed and analysed to calculate the number of appropriate information carrying words (AICW), as defined by Swinburn and colleagues (2005), and the syllable rate of speech production. Purely articulatory and dysarthria errors were not penalized when calculating AICW, but semantic and phonological errors were accounted for. For the Decision trials, the percentage of trials with correct target identification and the median reaction time for the responses were recorded (Table 1).

As post-stroke aphasia is a multidimentional disorder, the patients also performed a number of other assessments (Table 1) to capture the range of the deficit across several language and non-language neuropsychological domains: (i) Comprehensive Aphasia Test (CAT, Swinburn *et al.*, 2005) screening for language comprehension and production deficits. This included the cognitive subset of the CAT that taps into episodic memory, semantic memory, visual judgement, fluency, praxis and arithmetic; (ii) a brief version of the Raven’s Progressive Matrix Test (Arthur and Day, 1994); (iii) a modified version of the quantitative analysis of speech production based on patient’s narrative description of the Cinderella story (spontaneous speech) (Saffran *et al.*, 1989; Rochon *et al.*, 2000). The test of spontaneous speech allowed for a detailed assessment of narrative speech production ability taking into account information-carrying capacity of utterances, syntax and grammar without penalizing dysarthric errors (Geranmayeh *et al.*, 2016). The controls performed the cognitive section of the CAT, spontaneous speech production and the Raven’s Progressive Matrix Test on one occasion only.

A principal component analysis of the above measures at the early time (Time 1) was performed to determine the latent variable structure underlying correlations between all these scores (Supplementary Table 2). Based on this principal component analysis, the main behavioural measure of interest was identified as the in-scanner language measure. The language outcome was defined as the raw score at ∼4 months post-ictus, and the language recovery was defined as improvement in the scores over time (Time 2 − Time 1).

### Functional MRI procedure

Functional MRI data were obtained on Siemens Magnetom Trio 3 T scanner using gradient-echo sequences (36 slices acquired in an interleaved order; resolution: 3.5 × 3.5 × 3.0 mm; field of view: 225 × 225 × 108 mm; repetition time, 10 s; acquisition time, 2 s; echo time 31 ms; flip angle, 90°). A 1 mm^3^ T_1_-weighted image and field maps (used to reduce field inhomogeneity) were also acquired.

### Functional MRI paradigm

A ‘sparse’ functional MRI design (Hall *et al.*, 1999) was used to minimize artefacts associated with overt speech (Gracco *et al.*, 2005). Tasks were performed in response to specific visual stimuli during 7-s epochs, and terminated by the display of a fixation cross. Functional imaging data were acquired 1 s later, over 2 s. Each of the two functional MRI sessions after the stroke (early after stroke = Time 1, and 4 months after stroke = Time 2) included three runs. Each run consisted of four conditions; (i) 20 speech trials; (ii) 16 count trials; (iii) 16 decision trials; and (iv) 15 rest trials. Each condition was presented in blocks of two or four trials. Each of the four decision blocks were preceded by an instruction page that was displayed for 10 s.

During the Speech task, all participants were required to define nouns. These were presented in the form of coloured picture stimuli (Snodgrass and Vanderwart, 1980; Rossion and Pourtois, 2004). In total 120 pictures were chosen and matched across each of the runs with respect to imageability, concreteness, familiarity, and frequency based on measures derived from the Medical Research Council psycholinguistic database (Wilson, 1988). A different picture was displayed for 7 s at each trial. The participants were instructed to generate as much verbal information pertaining to the given object as possible. An example of a patient’s response to the picture of a spoon was ‘Spoon, (pause) it’s (pause) eat soup’. For the Count trials, participants counted up from 1 at a rate of 1/s upon seeing the prompt ‘1…’. During the Rest trials, they saw a fixation cross throughout the trial. For the Decision trials, they were required to press a button on seeing a target (a blue square) and make no response for a distractor (an orange circle), both of which were presented in equal frequencies in a random order. The participants received training before the scan.

### Image analysis

Data were preprocessed with the FMRIB Software Library (www.fmrib.ox.ac.uk/fsl). Preprocessing steps included: motion-correction; non-brain voxels removal; spatial smoothing (8 mm full-width at half-maximum Gaussian kernel); and high-pass temporal filtering. The six motion parameters derived from the motion-correction step, were included as regressors for each functional MRI run. Registration of functional MRI images to high resolution structural images was carried out by Boundary-Based Registration (Greve and Fischl, 2009) and fieldmap-based EPI distortion correction. The high resolution structural images were registered to the Montreal Neurological Institute standard space (MNI 152) using FMRIB’s Linear Image Registration Tool. A manually delineated binary inverted lesion mask was included to downweight the influence of the lesion in the registration and thus minimize distortions associated with the registration of brain with infarcts.

**Figure 2:**
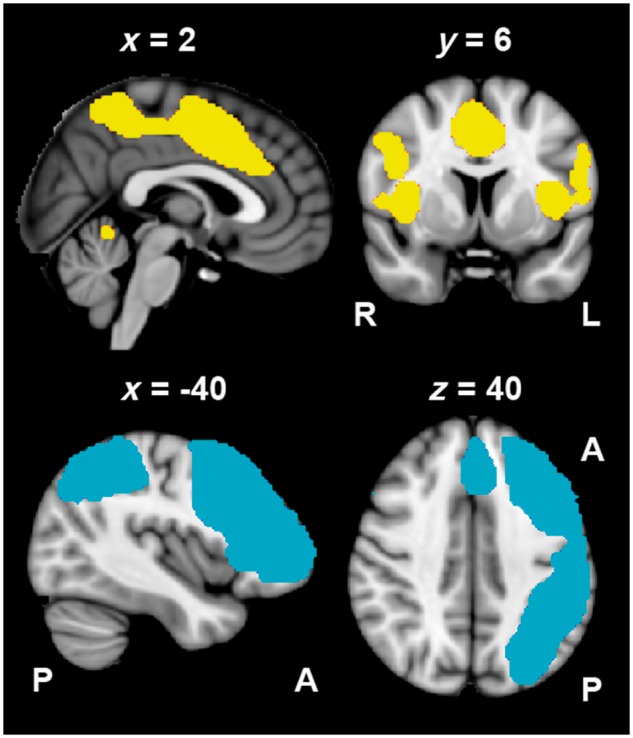
**Standard T_1_-weighted anatomical slices overlaid with a mask of domain-general brain activity within cingular-opercular network (*top*) and a left-lateralized frontal-parietal-temporal language-specific network (*bottom*).** The masks were obtained from previous analyses of control participants performing the same functional MRI task (Geranmayeh *et al.*, 2014b), and were used to calculate the lesion load in each network. R = right; L = left; A = anterior; P = posterior.

Lesion volume was calculated in standard space. As well as calculating gross lesion volume, the percentage damage to domain-general cingular-opercular network (Fig. 2, top) and a language-specific left-lateralized frontal-temporal-parietal network were also calculated for each patient (Fig. 2, bottom). These masks were obtained from previous analyses of the functional MRI data from the control participants performing the same paradigm (Geranmayeh *et al.*, 2014*b*). The cingular-opercular network included correlated activity within bilateral frontal operculum/anterior insular cortex and preSMA/dACC. Activity in this network (from a multivariate ICA) has been previously found to be increased during the non-verbal decision task compared to a relatively effortless speech production task in control participants (see Component 7, Fig. 3 in Geranmayeh *et al.*, 2014*b*). The left-lateralized frontal-temporal-parietal network showed activity that was greater for speech production at sentence-level in contrast to rest and in contrast to the counting or non-verbal Decision tasks (see Component 4, Figure 3 in Geranmayeh *et al.*, 2014*b*).

Individual functional MRI data were analysed in general linear models (GLMs) in SPM12, modelling the combination of the three runs for the three conditions over the two time points with the addition of nuisance variables to account for head movement and noise using a fixed effects model. Translations and rotations were captured in the *x*, *y* and *z* plane. Contrast images of interest depicting parameter estimates for the experimental predictor functions were collected from the first-level model and used in the group-level analyses.

At the individual subject level, parameter estimates for each of the three task conditions (Speech, Count, and Decision) were generated relative to the Rest baseline. These were collated and examined voxel-wise at the group level using a full factorial design in SPM12, in which the within subject factors were session (Time 1 versus Time 2) and task conditions (Speech, Count, and Decision). Data were collapsed across runs for each session and language measures and recovery were included as the covariate in the design.

The group-level effects were examined using a random effects analysis that used familywise error (FWE) cluster correction for the whole brain volume at *P* < 0.05. This analysis was restricted to a volume of the brain in which no patient had lesions. This meant a large proportion (290 cm^2^) of the left hemisphere, mainly in the territory of the left middle cerebral artery, was excluded from the analysis. This minimizes the confounding effects of heterogeneous lesion morphology in the patients.

To identify voxels that were involved in general cognition as opposed to language *per se*, the decision task was contrasted against the other two (Count and Speech), collapsed across both sessions. The midline voxel with highest activity during the Decision task was chosen as a functional region of interest. A measure of the mean activity of this region of interest during the Speech task was entered into a repeated measures ANOVA with Session as the within-subject factor and Language recovery (Time 2 – Time 1) as the between-subject covariate. The relationship between the activity in this region and recovery was further examined with correlation analyses and generalized linear models in SPSS.

Finally a confirmatory whole brain unconstrained voxel-wise analysis (excluding the lesioned volume) was performed to determine the areas of activity that correlated with the magnitude of the recovery of the language measure. Data from both time points were included in the model. Again, this was FWE cluster corrected for the whole brain volume excluding voxels of the lesion mask.

## Results

### Behavioural results

All reported *P*-values are two-tailed unless stated otherwise. The scores for the behavioural assessments are presented in Table 1. At the time of early assessment, patients had significantly reduced performance compared to the healthy participants on measures of spontaneous speech production, verbal fluency, and in-scanner language measure based on the Speech task performance. They also had poorer performance on the cognitive measure, Ravens Matrix and reaction time, although overall accuracy during the Decision task was not different to control subjects (Table 1). A repeated measures ANOVA on data from the patients showed a significant effect of session, suggesting that the patients had on average substantially recovered to some degree over the two time points [*F*(1,25) = 30.6, *P* < 0.001]. This improvement was seen across all assessments except accuracy on the Decision task (*post hoc* paired *t*-test, Table 1).

### Early predictors of language outcome and language recovery 4 months after stroke

Several demographic and stroke characteristics as well as scores set out in Table 1, were examined to identify potential confounding factors. Cognitive score (*r* = 0.603, *P* = 0.001), language measure (*r* = 0.826, *P* < 0.0001), and accuracy on the Decision task (*r* = 0.399, *P* = 0.039) during the early phase, positively correlated with language performance at 4 months. Reaction time for the decision trials had a negative relationship (*r* = −0.337, *P* = 0.04 one-tailed), with faster participants in the early scan, having a better language outcome at 4 months.

Total lesion volume (*r* = −0.555, *P* = 0.003) and more specifically percentage damage to a large left-lateralized frontal-temporal-parietal language network (*r* = −0.462, *P* = 0.015) and the domain-general cingular-opercular network (*r* = −0.438, *P* = 0.022) all negatively correlated with language outcome at 4 months (see Fig. 2 for representations of these two networks). Total years of education (*r* = 0.485, *P* = 0.01) had a positive correlation with language outcome at 4 months, while age (*P* = 0.54) and hours of speech therapy (*P* = 0.23) had no correlation.

When performing a multiple regression model with Language measure at Time 2 as the dependent variable and all the above behavioural measures, lesion characteristics and demographic factors as the independent variable, the model accounted for 72% of the variance in the language outcome at 4 months [*R^2^* = 0.72, *F*(10,16) = 7.67, *P* < 0.001]. Only early language measure performance (beta = 0.767, *P* = 0.002) and age (beta = −0.257, *P* = 0.047) were the overall significant predictors of language outcome at 4 months after stroke.

A similar regression model, this time with language recovery (Time 2 − Time 1) as the dependent variable, accounted for 63% of the variance in language recovery [*R^2^* = 0.63, *F*(10,16) = 4.95, *P* = 0.02]. Again, only early language performance (beta = −0.813, *P* = 0.002) and age (beta = −0.306, *P* = 0.047) were the overall significant predictors of language recovery over time.

### Domain-general non-verbal activity in the midline frontal cortex

**Figure 3:**
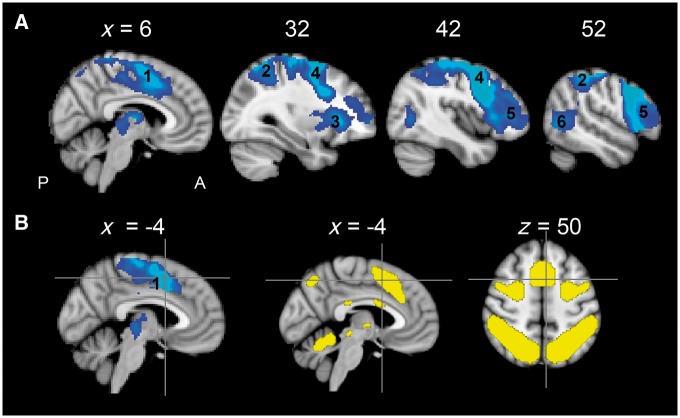
**Domain-general brain activity. (A)** Standard T_1_-weighted anatomical slices overlaid with domain-general brain activity showing areas of activity greater for the non-verbal decision task compared to the two verbal tasks (Count and Speech). Voxel-wise corrected at *P* < 0.05, then FWE cluster-wise corrected at *P* < 0.05. The numbered regions are as follows: (1) dACC and adjacent mid-cingulate cortex and preSMA; (2) superior parietal lobule extending to supramarginal gyrus and angular gyrus; (3) right anterior insular cortex; (4) precentral gyrus extending into middle frontal gyrus; (5) right inferior frontal gyrus; and (6) right posterior middle temporal gyrus. There was also bilateral thalamic activation. See Table 2 for coordinate details. (**B**) The cross hair corresponds to the midline peak activation voxel (*x*, *y*, *z* = −4, 8, 50) forming the basis of subsequent analyses in Fig. 4. The peak area of activity is marked over the activation pattern from the current analysis in blue, and over the domain-general brain regions more commonly known as the multiple demand cortex in yellow (Duncan and Owen, 2000; Fedorenko *et al.*, 2013).

To identify voxels that were involved in domain-general cognition, the decision task was contrasted against the other two (Count and Speech), collapsed across both sessions, with cluster correction for multiple comparisons (voxel-wise thresholded at *P* < 0.05, then FWE whole brain cluster-wise corrected at *P* < 0.05). As expected, a large cluster was evident along the midline extending back from the dorsal anterior cingulate cortex into mid-cingulate cortex, preSMA and the SMA with peak voxel within the preSMA (Fig. 3A). Activation was also evident within the precentral gyrus, right dorsolateral prefrontal cortex within the inferior frontal gyrus and the right parietal cortex and temporal parietal junctions. No activity was evident in the left homotopic regions as the lesioned volume was masked out in the analysis, to include only the non-lesioned brain across all participants. Parameter estimates were extracted from the peak voxel in the midline cluster, located within the preSMA (*x*, *y*, *z* coordinates: −4, 8, 50) for further analysis in relation to the behavioural measures (Fig. 3B, blue). Overlap of this coordinate over the set of brain regions known as multiple demand cortex (Duncan and Owen, 2000; Duncan, 2010) confirmed that this region is involved in domain-general cognitive processing (Fig. 3B, yellow).

**Table 2:** Coordinates for each local maxima within significant clusters of activation as shown in Figs 3 and 5

	Size (voxel number/cm^3^)	Z	MNI coordinates *(x, y, z)*	Harvard–Oxford cortical structural atlas
**Domain-general activity (** **Fig. 3** **)**				
1	19 566/157	5.59	42,8,32	Middle frontal gyrus, and precentral gyrus extending anteriorly into right middle frontal gyrus, inferior frontal gyrus and insular cortex. Extending posteriorly to superior and inferior parietal lobule.
Local maxima		3.56	0,6,34	Presupplementary motor cortex extending into right and left paracingulate cortex and anterior cingulate cortex
		3.18	56,−58,6	Right posterior middle temporal gyrus
**Correlation with language recovery (** **Fig. 5** **)**				
1	3008/24	5.74	2,−6,66	Bilateral supplementary motor cortex extending anteriorly to preSMA, bilateral paracingulate cortex, dorsal mid-cingulate cortex
2	417/3.3	2.84	50,−6,44	Right precentral gyrus extending to postcentral gyrus
3	258/2.1	2.46	62,−34,6	Right posterior superior temporal gyrus

### Presupplementary motor area/dorsal anterior cingulate cortex activity and language recovery over time

The peak voxel (*x*, *y*, *z* coordinates: −4 , 8, 50) from the analysis above (Fig. 3B) was chosen as the target functional region of interest for a region that demonstrated a sensitivity to general non-verbal cognitive demands. A one-sample *t*-test against 0 showed on average no significant activation in this region during the Speech task (*t* = −1.517, *P* = 0.141). The peak region is within the preSMA but the activation cluster extends into the dACC.

To determine the role of this preSMA/dACC region of interest in language recovery, its activity during the Speech task alone was extracted and entered into a repeated measures ANOVA with Session as the within-subject effect and Language recovery (change in score between Time 1 and Time 2) as the between-subject factor. There was no main effect of session [*F*(1,25) = 1.527, *P* = 0.228] and no interaction of Session × Recovery [*F*(1,25) = 1.075, *P* = 0.310]. Critically though, there was a significant main effect of Recovery [*F*(1,25) = 10.603, *P* = 0.003].

A generalized linear model with robust estimation was performed in SPSS with recovery as the dependent variable. The main predictor of interest was the activity in the preSMA/dACC averaged across both the early and the late sessions. In a second model, lesion volume was added as an additional predictor. Finally, a third model included preSMA/dACC activity, lesion volume, age, and the early language score as predictors. The latter two factors were included in the third model as they were identified as significant predictive factors in the above reported behavioural analyses. All three models were significant (*P* < 0.002; Table 3, top three models). The preSMA/dACC activity level was a significant predictor of recovery (*P* = 0.005). Specifically, for every extra point increased in activity in the preSMA/dACC, recovery was 1.02 [95% confidence interval (CI), 1.01–1.04] times greater (Table 3, Model 1). This relationship remained significant (*P* = 0.002) when adding in lesion volume (Table 3, Model 2). Indeed, lesion volume was not a significant predictor (*P* = 0.45). Finally, the relationship remained significant (*P* = 0.02) when adding in early language score and age, both of which had a significant negative relationship with recovery (Table 3, Model 3). Similar results were obtained when using language outcome at ∼4 months as the dependent variable (Table 3, bottom).

**Table 3:** Generalized linear model analyses

Predictor	B	df	Significance	Exp(B)	CI Exp(B)
**Dependent variable = language recovery (Time 2 − Time 1)**					
Model 1 (*P < *0.001)					
preSMA/dACC	0.021	1	0.005	1.02	1.01–1.04
Model 2 (*P < *0.002)					
preSMA/dACC	0.018	1	0.002	1.02	1.01–1.03
Lesion volume	<0.000	1	0.451	1.00	1.00–1.00
Model 3 (*P < *0.0001)					
preSMA/dACC	0.010	1	0.022	1.01	1.00–1.02
Lesion volume	−<0.000	1	<0.739	1.00	1.00–1.00
T_1_ language measure	−0.364	1	<0.001	0.69	0.59–0.83
Age	−0.034	1	0.003	0.97	0.95–0.99
**Dependent variable = Language outcome at Time 2**					
(*P < *0.0001)					
preSMA/dACC	0.010	1	0.022	1.01	1.00–1.02
Lesion volume	−<0.000	1	<0.739	1.00	1.00–1.00
T_1_ language measure	0.636	1	<0.001	1.89	1.59–2.24
Age	−0.034	1	0.003	0.97	0.95–0.99

Activity in the preSMA/dACC was a significant predictor of recovery and outcome taking into account effects of confounding variables lesion volume, age and initial language score. B = coefficient estimates of the regression; Exp(B) = exponentiated values of the coefficients (more informative); CI = 95% Wald confidence interval for Exp(B).

**Figure 4:**
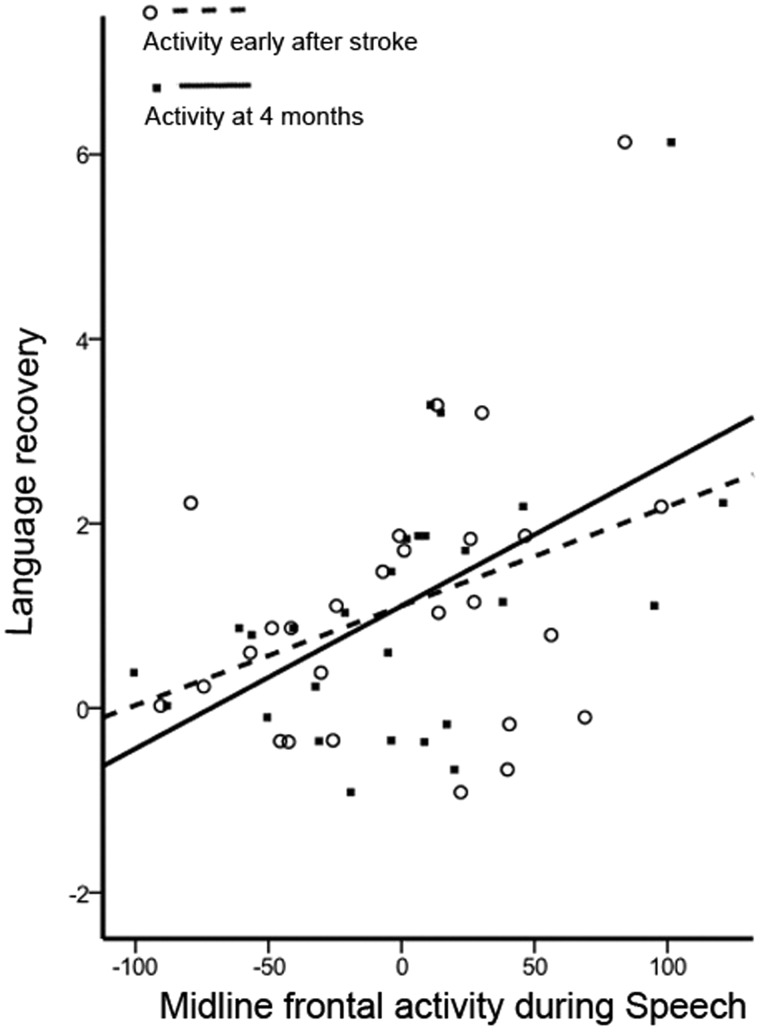
**Relationship between midline-frontal (preSMA/dACC) activity and language recovery over time.** Different correlation plots are shown based on the activity in this region during the early scan (dotted line) and the late scan at 4 months after stroke (solid line). There was a significant positive relationship between activity at each time point and level of language recovery.

Further testing confirmed that the activity in the peak voxel of midline frontal cortex in the preSMA/dACC, during the early scan (*r* = 0.363, *P* = 0.03 one-tailed) and late scan (*r* = 0.538, *P* = 0.004) when performing the Speech task were both correlated positively with degree of language recovery (Fig. 4). The positive correlation with language recovery was only observed for activity within the preSMA/dACC region during the Speech task, and not for the activity when participants performed other tasks (e.g. the non-linguistic Decision task).

### Whole-brain correlation with language recovery

**Figure 5:**
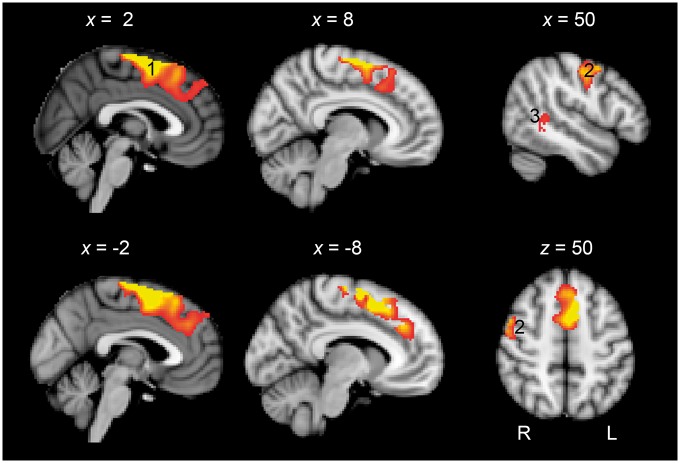
**Standard T_1_-weighted anatomical slices overlaid with activity correlating with language recovery.** A large cluster of activity was observed in the preSMA extending to dorsal mid-cingulate cortex and dACC (1). Activity was also observed in the right pre- and post-central gyri (2) and right posterior superior temporal gyrus. (Rendered at *P* < 0.05 with 200 voxel extent. Peak voxel whole brain significant at *P* < 0.001 FWE corrected). See Table 2 for coordinate details.

The relationship between language recovery and midline frontal cortex brain activation was examined further using an unconstrained whole brain voxel-wise analysis (cluster rendered at *P* < 0.05 with 200 voxel extent). A single cluster was evident that encompassed the dACC and extended back through the preSMA and SMA (Fig. 5). The peak voxel within this cluster was significant at *P* < 0.001 FWE corrected for the whole brain volume.

## Discussion

This longitudinal functional MRI study examined how activity within a domain-general brain region relates to the magnitude of recovery of language after a left hemisphere stroke. The preSMA/dACC was proposed in advance as a region with domain-general cognitive function and it was localized in the current study by its robust activation during a decision-making task relative to language tasks. The level of activity in this region when patients attempted to speak was positively correlated with the level of spontaneous recovery of speech production after stroke.

To account for the contribution of behavioural factors to the language outcomes and recovery, we first identified these factors amongst several potential measures in separate multiple regression analyses. The early language performance—a marker of initial aphasic stroke severity—and age were identified as the overall significant predictors of both the language performance at 4 months and the recovery over time in keeping with previous studies (Lazar *et al.*, 2008; Knoflach *et al.*, 2012; Plowman *et al.*, 2012). Therefore, we accounted for these two factors, along with lesion volume, in the subsequent analyses. The activity in the preSMA/dACC was a significant predictor of recovery of language in a model that included lesion volume, age and initial aphasic severity (as measured by in-scanner language performance) as additional confounds. These results accord closely with the hypothesis that the preSMA/dACC is involved in the recovery process.

The contribution of domain-general brain regions in recovery from aphasic stroke has previously been proposed in the neuropsychological literature (Robertson and Murre, 1999; Purdy, 2002; Wiener *et al.*, 2004; Baldo *et al.*, 2005; Swinburn *et al.*, 2005; Fillingham *et al.*, 2006; Fridriksson *et al.*, 2006). More recently functional neuroimaging has provided neurobiological evidence to support the notion that language processes involve an interaction between language specialized and domain-general brain regions (Fedorenko, 2014; Fedorenko and Thompson-Schill, 2014; Geranmayeh *et al.*, 2014a, *b*). One network of brain regions that has been attributed to domain-general status is the cingular-opercular network. This network is defined by correlated activity in the dACC and adjacent preSMA and bilateral anterior insular and adjacent frontal operculum. This network is a subsystem of the multiple demand cortex volume that is associated with a particularly diverse range of cognitive demands (Duncan and Owen, 2000; Duncan, 2010, 2013). Notably, and of relevance to recovery after stroke, we have previously reported that this network is particularly active during intentional modes of learning (Doyon *et al.*, 1996; Toni *et al.*, 2001; Hampshire *et al.*, 2016), including when learning a pseudo-language consisting of novel word-picture associations (Sliwinska *et al.*, submitted for publication).

One cross-sectional study by Brownsett *et al.* (2014) specifically addressed the contribution of the dACC node of the cingular-opercular network to language outcomes in chronic aphasic patients. The activity in the dACC and adjacent superior frontal gyrus in the chronic phase during a language task was positively correlated with performance on an out-of-scanner overt picture description task. This was interpreted as showing increased activation of the cingular-opercular network during increased task difficulty in patients. Two further studies in chronic aphasic stroke patients have confirmed that (i) the activity in this network is upregulated when aphasic patients perform a language task (Geranmayeh *et al.*, 2016); and (ii) activity in the subregions of this network, including dACC and right insula cortex, are correlated with improvement after picture naming training (Raboyeau *et al.*, 2008). As far as we are aware the only longitudinal study that has suggested a link between activity in dACC/PreSMA region and short-term spontaneous recovery is that by Saur and colleagues (2006). They showed that the recovery of language function over the first 2 weeks after stroke shows a positive correlation with the change in activity in this region over the same period. The current study extends these works in four critical ways.

First, the current study was specifically designed to address the contribution of domain-general brain regions to language recovery and thus included a non-verbal functional MRI task that was used to identify the domain-general region of interest. Notably, the patients, as a group, did not activate this region significantly relative to rest during the speech production task itself, although age-matched healthy controls did (see region number 3, Figure 1 in Geranmayeh *et al.*, 2014*b*). This suggests a link between aphasic stroke and ability to engage this brain region during language processing, perhaps as a result of structural disconnection from upstream or downstream regions.

Second, we were able to show that activity level related positively to the magnitude of recovery whilst taking into account the additional effects of the initial severity of aphasia (based on the early language measure) and of age. These were selected to be the most significant confounding factors amongst a number of potential demographic, and stroke-related factors. Suggesting that a polymarker that is based on all three measures could potentially provide a subacute phase tool for approximating the subsequent recovery.

Third, we examined a different longitudinal time window to the abovementioned longitudinal study, namely from early after stroke (∼2 weeks) to 4 months after ictus. This is important because the first 2 weeks after stroke is a time that is prone to rapid changes in vascular reactivity that can confound the results. We have previously analysed the vascular reactivity of the current cohort and concluded that the changes in activity over the study time are not confounded by changes in vascular reactivity. This was made possible by including a breath-hold functional MRI paradigm at each functional MRI session (Geranmayeh *et al.*, 2015*b*).

Fourth, we were able to show that the absolute level of activation at each time point (i.e. during both the early and the late scan) had a positive predictive value of spontaneous recovery over the first 4 months after stroke; this predictive relationship is important because it means that a measure of domain-general functional activity early after stroke could potentially be used as a neuroimaging biomarker together with other factors to predict longer term outcome.

Our analyses focused on testing a specific prediction of our hypothesis that the preSMA/dACC is involved in the language recovery process. It is worth noting that we did not observe a relationship between recovery and activity within other parts of the cingular-opercular network, namely the bilateral anterior insula and frontal operculum. This may partly be a methodological effect as we excluded the majority of the left perisylvian region as this had been lesioned in a number of patients.

The observed relationship of reduced preSMA/dACC activity and recovery is likely to have relationship with brain structure. It could be due to (i) pre-existing pathology, for instance small vessel disease or undiagnosed neurodegenerative disease burden; (ii) a simple relationship as a direct result of stroke lesion volume; and (iii) a more complicated relationship as an indirect result of the stroke disrupting the inputs to this region. While this is an important question to address, it is beyond the scope of the current study and would be suited to a much larger scale study that allows for nuanced multivariate analyses. Nevertheless, we were able to show that the effect of preSMA/dACC on recovery does not have a simple relationship with the overall lesion volume.

Based on the results presented here, and the observation of increased cingular-opercular network activity during difficult language tasks (Geranmayeh *et al.*, 2014*a*, 2016) and during various modes of learning (Doyon *et al.*, 1996; Toni *et al.*, 2001; Hampshire *et al.*, 2016) including when learning a pseudo-language (Sliwinska *et al.*, submitted for publication), we believe that this network is likely to be a good target candidate for experimental therapeutics including neurostimulation. Within this network, the dACC/preSMA is a particularly attractive target because it is usually spared after aphasic stroke within the left middle cerebral artery territory. Thus, enhancing midline activity in dACC/preSMA, or the connectivity within the cingular-opercular network, could accelerate language recovery. It also is targetable even via non-invasive neurostimulation techniques, whereas other nodes of this network are less accessible. Indeed taking a network-based approach to stimulate functionally linked brain regions has been shown to improve outcomes after neurostimulation in various pathological states (Fox *et al.*, 2014) and in aphasia (Meinzer *et al.*, 2016). Moreover, we have reported in a parallel piece of research that transcranial magnetic stimulation of dACC/preSMA accelerates the learning of a novel pseudo-language consisting of psuedoword-picture pairs (Sliwinska *et al.*, submitted for publication). Given the broad cognitive role of this network, we believe that the therapeutic potential for this network could extend to recovery from various forms of brain damage. Testing this hypothesis forms the basis of our future work.

## Supplementary Material

Supplementary Data

## Abbreviation

preSMA: dACC
presupplementary motor area: dorsal anterior cingulate cortex
