# Supplementary material for: Domain-general subregions of the medial prefrontal cortex contribute to recovery of language after stroke
Source: Brain. 2017 Jun 27;140(7):1947–58. doi: 10.1093/brain/awx134 (PMC5903407; doi:10.1093/brain/awx134)
Supplement: Supplementary Data [file awx134_supp.zip › brain-2017-00295-File010.pdf]

Supplementary Material, Table 1: *List of patients*. All patients were right handed except that marked with *, who was aphasic after a left hemisphere infarct suggestive of left hemisphere dominance for language. Patient denoted with [+] had an additional small right sided. Patients had received variable amount of speech therapy (SALT). H, hypertension; Cl, hypercholesterolemia; Is, ischaemic heart disease; Ti, previous small cerebrovascular disease or transient ischaemic attacks; S, smoker; eS, ex-smoker; A; atrial fibrillation. Lesion location is in the left hemisphere unless stated otherwise: C, cortical; SC wm, subcortical white matter; SC gm, subcortical grey matter; I, insular; F, frontal; P; parietal, T; temporal; O, occipital. NIHSS, National Institute of Health Stroke Scale; T1, Early assessment after stroke (~ 2 weeks); T2, ~ 4 months after stroke.

	Sex	Age	Age at leaving education (years)	Thrombolysis	Lesion volume (cm^3)	Lesion Location	Cerebrovascular risk factors	Time of first scan (days post stroke)	Time of second scan (days post stroke)	Hours of speech and language therapy	NIHSS		Language measure		Cognitive measure		Percentage Correct Decision		Reaction time (seconds)	
											T1	T2	T1	T2	T1	T2	T1	T2	T1	T2
1	M	67	16	Y	165.6	C, SC wm (I, F, T, P)	eS, H, A, I	35	90	2	12	1	3.0	2.1	24	36	0.98	1.00	0.54	0.59
2	M	77	14	N	48.7	C, SC wm (F, I)	A, H	28	104	2	4	1	2.3	3.2	33	38	0.90	0.93	0.43	0.49
3	F	68	24	N	49.5	C (F) anterior circulation	A, H	30	154	10	2	0	2.8	4.9	33	38	0.93	0.92	0.53	0.45
4	F	46	12	N	10.3	C, SC wm (F, I)	eS	14	119	0	3	0	1.9	3.6	37	37	1.00	1.00	0.41	0.39
5	F	77	21	N	4.5	SC wm, SC gm (I)	S	11	114	0	2	0	5.0	5.2	38	38	1.00	0.99	0.50	0.48
6	M	50	21	N	54.8	C, SC wm (F, I O, P)	H, I, D, C	12	102	5	2	0	2.2	5.5	33	37	0.97	1.00	0.46	0.43
7	M	44	16	Y	20.5	SC wm, SC gm (F, I)	C, S	12	161	1.5	7	1	3.8	5.7	31	37	0.95	0.94	0.31	0.3
8[+]	M	46	16	N	48.7	C, SC wm, SC gm (F, P, T, O and right F)	D, H, C	15	200	4	1	1	3.1	3.4	33	32	0.96	0.96	0.38	0.36
9	M	76	25	Y	3.8	C, SC wm (P, I)	A, H	25	124	0	2	0	4.6	4.4	38	38	0.99	1.00	0.40	0.43
10[+]	M	60	21	N	12.6	SC wm (F), Ψ	D, H, T, C	10	127	0	2	1	5.1	6.1	38	38	0.97	0.94	0.40	0.33
11	M	56	14	N	46.5	C , SC wm (P, F, T)	D, H	17	96	10	5	1	0.7	1.8	27	34	0.52	0.99	0.32	0.41
12	M	57	18	Y	60.4	C, SC wm (P, F)	S	20	90	7	6	1	1.1	4.3	32	37	1.00	1.00	0.37	0.34

13	M	75	26	Y	36.4	C , SC wm (T, O) posterior circulation	C, I	16	101	0	1	0	7.2	7.1	36	36	0.98	0.97	0.37	0.35
14	M	65	16	Y	22.7	C, SC wm (F, I)	-	6	101	0	2	0	1.4	2.3	32	37	0.92	1.00	0.32	0.38
15 *	M	64	22	Y	64.3	C, SC wm (I, F, P)Ψ	C	6	89	7	13	2	1.7	4.0	35	38	1.00	1.00	0.42	0.37
16	M	64	24	N	25.4	C, SC wm (F, P)	A, H, C	12	96	18	1	0	6.5	6.5	33	38	1.00	1.00	0.37	0.36
17[+]	F	39	22	N	29.9	C, SC wm (F, I) Ψ	T	20	91	0	0	0	5.8	6.4	38	38	1.00	1.00	0.39	0.34
18	M	65	14	N	12.2	C, SC gm (T)	H, I, C, S	11	104	0	5	1	4.2	6.0	33	37	1.00	0.98	0.40	0.38
19	F	49	24	N	5.3	C (F)	-	18	88	0	0	0	6.1	7.6	38	38	0.99	1.00	0.35	0.35
20	M	53	22	N	5.6	C SC wm (F)	-	5	102	0	1	0	5.4	5.0	37	37	0.99	1.00	0.44	0.43
21	F	69	15	N	112.5	C, SC wm (T,P,I)	H, eS, D	9	87	4	4	1	2.2	3.0	36	38	0.99	1.00	0.88	0.51
22	M	54	15	N	35.8	C, SC wm (F)	I, H, C	14	99	0	1	0	2.4	3.5	30	32	0.92	0.96	0.45	0.44
23	F	53	14	Y	29.7	C, SC wm (F)	H , A	8	92	12	3	1	1.6	3.5	34	34	0.96	0.99	0.49	0.4
24	M	63	18	N	6.8	SC wm	C	7	90	0	1	0	6.4	6.1	38	38	0.97	1.00	0.36	0.43
25	M	50	17	N	9.7	C, SC wm (I, T)	D, H, C, S	20	101	0	0	0	9.3	8.9	38	38	0.99	1.00	0.33	0.33
26	F	48	21	Y	173.9	C, SC wm gm (F P O)	H, I	17	95	10	10	4	-3.7	2.5	19	36	1.00	0.99	0.47	0.44
27	F	62	21	Y	20.5	C , SC wm gm (P)	H	10	94	13	2	0	5.3	4.6	38	38	0.99	1.00	0.42	0.35
