# Supplementary material for: Domain-general subregions of the medial prefrontal cortex contribute to recovery of language after stroke
Source: Brain. 2017 Jun 27;140(7):1947–58. doi: 10.1093/brain/awx134 (PMC5903407; doi:10.1093/brain/awx134)
Supplement: Supplementary Data [file awx134_supp.zip › brain-2017-00295-File011.pdf]

Supplementary Table 2. Principal Component Analysis. In order to reduce the behavioral measures listed in Table 1 of the manuscript, into a smaller number of factors that best explained the variability in all the behavioral results, a Principal Component Analysis was performed on all the measures at Time 1. Three principal orthogonal components were generated with eigenvalues >1. The component loadings for each factor are summarized in the table below. The first factor accounting for 38% of the variance in the data loaded equally heavily on the In-scanner language measure as well as naming, reading, spontaneous speech, fluency and written comprehension. We chose the in-scanner language measure as the main measure of interest to relate to imaging parameters, as changes in its score most reliably underlie the fluctuations in functional activity during this task. Underlined values indicate the tests with high loading to each factor.

	Component		
	1	2	3
Rotation sums of squared loading (Measure of Eigenvalue)	5.2	4.0	1.4
% variance explained	38	29	10
Cognitive score	0.756	-0.038	0.188
**Spontanous Speech**	**0.836**	0.223	-0.231
**Verbal fluency**	**0.864**	0.035	-0.145
Spoken Comprehension	0.798	-0.441	0.322
**Written Comprehension**	**0.821**	-0.377	0.233
Repetition	0.752	0.378	-0.094
**Naming**	**0.883**	0.138	0.033
**Reading**	**0.881**	-0.048	-0.108
Spoken Picture description	0.781	0.296	-0.204
Ravens Matrix	0.73	-0.071	0.383
**Language Measure**	**0.849**	-0.062	-0.156
Syllable Rate	0.665	0.067	-0.528
Decision task - % correct response	0.414	0.393	**0.684**
Decision task - mean reaction time	-0.272	**0.826**	0.199
